# The Increased Circulating Plasma Levels of Vascular Endothelial Growth Factor in Patients with Type 1 Diabetes Do Not Correlate to Metabolic Control

**DOI:** 10.1155/2017/6192896

**Published:** 2017-03-21

**Authors:** Kailash Singh, Stellan Sandler, Daniel Espes

**Affiliations:** ^1^Department of Medical Cell Biology, Uppsala University, Uppsala, Sweden; ^2^Department of Medical Sciences, Uppsala University, Uppsala, Sweden

## Abstract

*Aim.* To characterize the plasma levels of vascular endothelial growth factor (VEGF) in type 1 diabetes mellitus (T1D) and its relation to both present and historical metabolic control and microvascular complications.* Methods.* Plasma levels of VEGF and routine clinical parameters were analyzed in 115 patients with long-standing T1D and 45 healthy controls (HC). All patients were under clinical routine diabetes treatment at Uppsala University Hospital.* Results.* The plasma levels of VEGF were increased by 37% in patients with T1D when compared to HC (18.2 ± 0.8 versus 13.2 ± 1.0 pg/ml, *p* < 0.001). The levels of VEGF correlated to insulin needs and BMI but not to present or historical metabolic control. The levels of VEGF were similar in patients with T1D and microvascular complications (microalbuminuria and retinopathy) when compared with patients without microvascular complications. Historical HbA1c levels were found to be the best predictor for present metabolic control.* Conclusion.* Circulating plasma levels of VEGF do not correlate to present or historical metabolic control in long-standing T1D and the levels are not affected by the presence of microvascular complications.

## 1. Introduction

Type 1 diabetes mellitus (T1D) is one of the most common chronic diseases in children and young adults in the western world. Despite modern insulin treatment T1D is associated with a number of chronic complications and the life expectancy of patients with T1D is reduced by more than 10 years [1]. In the Diabetes Control and Complications Trial (DCCT) a link between glycemic control and the risk of diabetic complications was established [2]. In a recent publication from the original DCCT it was reported that the T1D patients who received intensive insulin treatment during the 6.5 years of the trial still had a reduced risk of complications 30 years later [3]. It therefore becomes obvious that not only the present glycemic control but also the historical metabolic control is of great importance for the development of micro- and macrovascular complications. In previous studies of patients treated under routine diabetes care the historical HbA1c levels have been shown to be associated with both the development of and progression of nephropathy and retinopathy [4]. Most of the complications in T1D are related to negative effects on the vascular system due to hyperglycemia although the exact underlying mechanisms are not fully understood. The most common diabetic complication is retinopathy and already after seven years of disease duration 50% of T1D patients display retinopathy to some extent [5]. However, one of the most feared complications is diabetic nephropathy since it can lead to end-stage renal failure which requires dialysis or renal transplantation [6].

Vascular endothelial growth factor (VEGF) was first found to be secreted from tumor cells and described as “vascular permeability factor” due to the increased permeability of vessels in animals bearing ascites tumors [7]. VEGF has since then been extensively studied in the field of tumor biology but also in other pathological conditions due to its importance in normal physiology [8]. In experimental studies of diabetes hyperglycemia has been shown to induce vascular hyperpermeability and increase blood flow, but these effects can be ameliorated by blocking VEGF via neutralizing antibodies [9]. In addition, it has been demonstrated in a number of experimental studies that increased levels of glucose increase both the gene expression and protein content of VEGF in vascular smooth muscle cells [10, 11] and retinal epithelial cells [12]. VEGF has, in a number of reports, also been proposed to correlate to microvascular complications, such as nephropathy and retinopathy [13–18] in patients with T1D. The circulating levels of VEGF have been reported to be increased in patients with T1D when compared to healthy controls [14, 17]. Moreover, a positive correlation between the average HbA1c levels of the most recent year and the levels of VEGF has been reported [14]. However, there have to the best of our knowledge been no reports on the importance of VEGF and historical metabolic control and the association with microvascular complications.

## 2. Study Participants, Materials, and Methods

The study was approved by the regional ethical board of Uppsala County and conducted in accordance with the declaration of Helsinki as revised 2000. All study participants were given oral and written information and signed a consent form before inclusion. In total 115 patients with T1D, all with >10 years of disease duration, and 45 healthy controls (HC) were included. There were no differences in age, BMI, or gender distribution between the two groups, for descriptive data (Table 1). All patients were under treatment and continuous standard routine diabetes care at Uppsala University Hospital. All blood samples were collected under standardized conditions after overnight fasting. Plasma was separated by centrifugation of EDTA-tubes and immediately frozen and stored at −80°C. The samples were not thawed prior to analysis. VEGF was analyzed in plasma by mesoscale (Meso Scale Diagnostics, Rockville, MA) according to the manufacturer's protocol. All other biochemical parameters were analyzed according to the clinical routine at the central clinical laboratory of Uppsala University Hospital. Microalbuminuria was defined as urine albumin > 30 mg/l but < 300 mg/l. Estimated glomerular filtration rate (GFR) was calculated based on creatinine levels, age, and gender using the Modification of Diet in Renal Disease (MDRD) formula [19]. Clinical parameters were assessed on the same day as blood sampling. Historical clinical data for T1D patients were retrieved from patient journals and the Swedish National Diabetes Registry (NDR). Average historical HbA1c levels were calculated as a mean over the last 10 years. Screening for retinopathy was performed within the last 12 months.

Statistical analysis was performed using Graph Pad Prism version 6.03. An unpaired two-tailed *t*-test based on ranks (Mann–Whitney) was used to compare differences between two groups for data that were not normally distributed and a two-tailed unpaired Student's *t*-test was used for normally distributed data. Normality distribution was determined using D'Agostino-Pearson Omnibus normality test. All correlations were calculated using nonparametric Spearman correlation since the levels of VEGF were not normally distributed. All *p* values <0.05 were considered statistically significant. All values are given as means ± SEM.

## 3. Results

**Figure 1:**
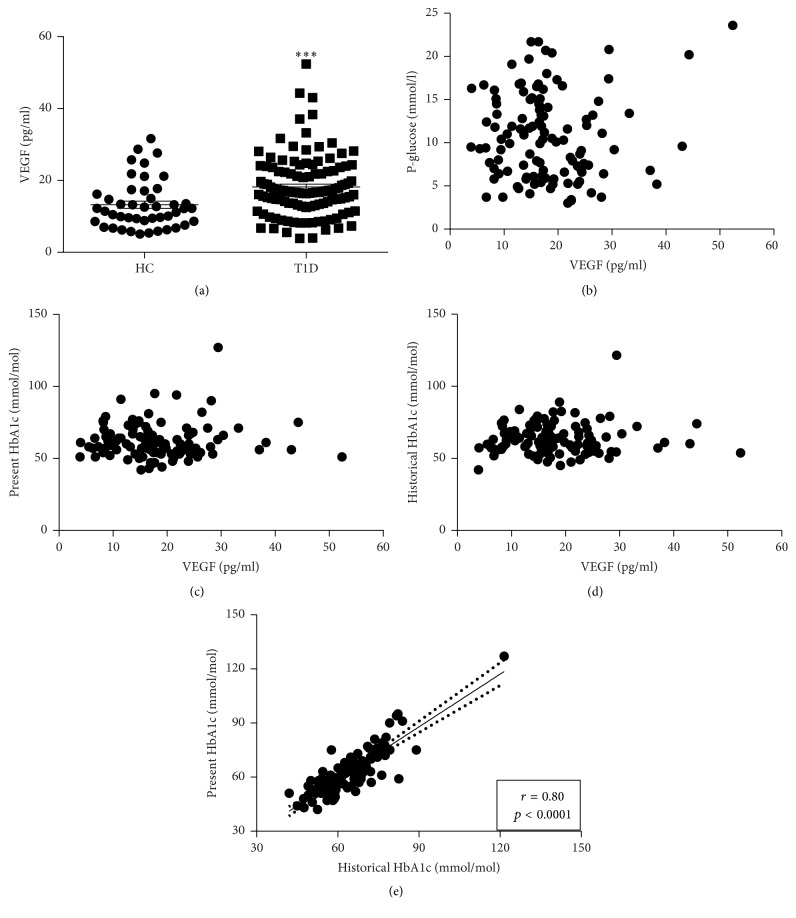
Increased levels of plasma VEGF in type 1 diabetes do not correlate to metabolic control. (a) The circulating levels of VEGF in plasma were increased in patients with long-standing (>10 years) type 1 diabetes (T1D, *n* = 115) when compared to matched healthy controls (HC, *n* = 45). In T1D patients there was no correlation between the plasma levels of VEGF and fasting plasma glucose (b), present HbA1c levels (c), or the average historical HbA1c levels from the last 10 years (d). However, there was a positive correlation between average historical and present metabolic control (e). An unpaired two-tailed *t*-test based on ranks (Mann–Whitney) was applied in (a) since VEGF was not normally distributed. Values are given as mean ± SEM. All correlations were calculated using nonparametric Spearman correlation. *∗∗∗* denotes *p* < 0.001.

**Figure 2:**
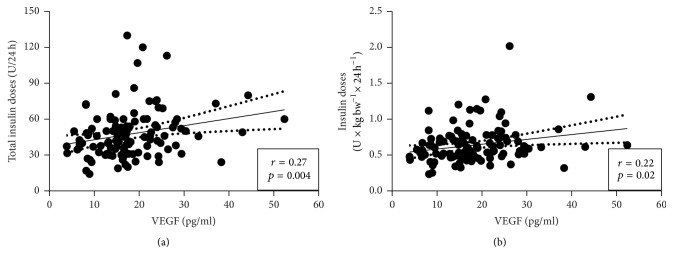
Plasma levels of VEGF correlate to exogenous insulin needs in patients with type 1 diabetes. The circulating plasma levels of VEGF were correlated to both the total exogenous insulin doses (a) and exogenous insulin doses adjusted for body weight (b) in patients with long-standing (>10 years) T1D (*n* = 115). Correlations were calculated using nonparametric Spearman correlation.

The plasma levels of VEGF were increased by 37% in patients with T1D when compared to HC (18.2 ± 0.8 versus 13.2 ± 1.0 pg/ml, *p* < 0.001) (Figure 1(a)). The levels of VEGF did not correlate to blood glucose levels (*p* = 0.38), present HbA1c levels (*p* = 0.37), or average historical HbA1c levels (*p* = 0.75) in patients with T1D (Figures 1(b)–1(d)). However, a strong positive correlation was observed between the present and average historical HbA1c levels (*r* = 0.86, *p* < 0.0001) (Figure 1(e)), which was also observed for the present and historical 5-year value (*r* = 0.52, *p* < 0.0001). There was a positive correlation between levels of VEGF and total insulin doses (U/24 h) (*p* < 0.01, *r* = 0.27) and insulin doses corrected for body weight (U × 24 h^−1^  × kg^−1^) (*p* < 0.05, *r* = 0.22) (Figures 2(a) and 2(b)). Measures of kidney function (creatinine and glomerular filtration rate (GFR)) did not correlate to the levels of VEGF (data not shown). There was a tendency towards a positive correlation between levels of VEGF and body weight (*p* = 0.0501) and a positive correlation to BMI (*p* < 0.05, *r* = 0.22) in subjects with T1D. The levels of VEGF did not correlate with age, age at onset of T1D, or disease duration (data not shown). Among the subjects with T1D there was no difference in the levels of VEGF between men and women (*p* = 0.64).

**Figure 3:**
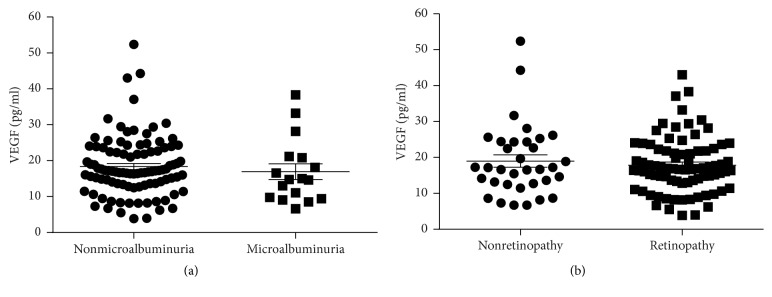
The levels of plasma VEGF are similar in patients with type 1 diabetes and microvascular complications when compared to patients without complications. Among the patients with T1D (*n* = 115), *n* = 17 had microalbuminuria (defined as urine albumin between 30 and 300 mg/l) but the levels of plasma VEGF were similar as compared to patients without microalbuminuria (a). Also, patients with retinopathy (*n* = 81) had similar levels of plasma VEGF when compared to patients with a normal eye exam. An unpaired two-tailed *t*-test based on ranks (Mann–Whitney) was applied in (a) since VEGF was not normally distributed. Values are given as mean ± SEM.

Out of the total 115 patients with T1D, *n* = 17 (15%) had microalbuminuria. However, there was no difference in the plasma levels of VEGF (17 ± 2.2 versus 18.4 ± 0.8 pg/ml, *p* = 0.29) between the two groups (Figure 3(a)). In fact, the levels of VEGF in patients with microalbuminuria were similar to those in HC (17 ± 2.2 versus 13.2 ± 1.0 pg/ml, *p* = 0.12). Interestingly, the total insulin doses were lower (42 ± 5 versus 49 ± 2 U/24 h, *p* < 0.05) among the patients with microalbuminuria, which was also true when the insulin doses were corrected for body weight (0.54 ± 0.05 versus 0.66 ± 0.03 U × 24 h^−1^  × kg^−1^, *p* < 0.01). The present (64 ± 3 versus 62 ± 1 mmol/mol (8 ± 0.3 versus 7.8 ± 0.1%), *p* = 0.36) and historical HbA1c levels (67 ± 1 versus 64 ± 1 mmol/mol (8.3 ± 0.1 versus 8.0 ± 0.1%), *p* = 0.14) were similar between the two groups. Creatinine and GFR did not differ between the two groups (data not shown). However, the systolic blood pressure was increased in patients with T1D and microalbuminuria (131 ± 3.3 versus 121 ± 1.3 mmHg, *p* < 0.01).

The majority of the patients with T1D had retinopathy (*n* = 81, 70%) but there was no difference in plasma levels of VEGF when compared with patients without retinopathy (17.8 ± 0.9 versus 19.0 ± 1.7 pg/ml, *p* = 0.82) (Figure 3(b)). The levels of VEGF were however increased when compared with HC (17.8 ± 0.9 versus 13.2 ± 1.0 pg/ml, *p* = 0.0003). As for microalbuminuria the insulin needs were lower in patients with retinopathy as compared to those without retinopathy, both when expressed as total insulin doses (45 ± 2 versus 53 ± 4 U/24 h, *p* < 0.05) and when corrected for body weight (0.58 ± 0.02 versus 0.78 ± 0.06 U × 24 h^−1^  × kg^−1^, *p* < 0.001). There was no difference in the present (63 ± 1 versus 60 ± 2 mmol/mol (7.9 ± 0.1 versus 7.6 ± 0.2%), *p* = 0.20) or historical HbA1c levels (66 ± 1 versus 62 ± 2 mmol/mol (8.2 ± 0.1 versus 7.8 ± 0.2%), *p* = 0.13). The mean systolic blood pressure was higher, although within the treatment range, in patients with T1D and retinopathy (124 ± 1 versus 118 ± 2 mmHg, *p* = 0.025).

Sixteen patients with T1D (14%) had both microalbuminuria and retinopathy, whereas 29 (25%) had neither microalbuminuria nor retinopathy. Even when comparing the plasma levels of VEGF between these two groups no difference was observed (17.1 ± 2.3 versus 18.6 ± 1.9 pg/ml, *p* = 0.55).

## 4. Discussion

In line with previous reports [14, 17] we found that the circulating levels of VEGF are increased in patients with T1D when compared to HC. In contrast to previous reports [14, 16], we did not observe any correlation between the present HbA1c levels and VEGF. However, in most previous reports VEGF have been analyzed in serum [14–17]. The major difference between plasma and serum is the contribution of VEGF released from platelets and leukocytes in serum. The many pitfalls associated with analyzing circulating VEGF are described in a review by Jelkmann and the overall conclusion is that VEGF should be analyzed in plasma [20]. Also, the use of anticoagulant when collecting the plasma can affect the results [21, 22]. In addition, not only the anticoagulant but also the analyzing center, centrifuge, and analyzing method have been found to be independent confounders for the measurement of circulating plasma levels of VEGF [22]. With that in mind it is therefore difficult to compare different studies reporting on circulating levels of VEGF since there are so many aspects that can influence the results. In addition, it should be noted that both the studies by Chiarelli et al. [14] and Seckin et al. [16] were performed in a pediatric population of patients with a shorter disease duration (two years). Despite the known impact of historical metabolic control on diabetic complications [3], we did not observe any correlation between levels of VEGF and average historical HbA1c levels. Nor when correlations were calculated for single HbA1c levels from each year over the last 10 years was any correlation observed (data not shown). Our study material consists of patients under routine clinical diabetes care without any specific interventions strategy and we observed, as expected, that the best predictor for present HbA1c levels was the average historical HbA1c levels. In fact, it has previously been shown in a study of more than 600 T1D patients that even after an intensified insulin treatment and structured patient education the historic HbA1c level is one of the best predictors for long term metabolic control [23].

Interestingly, we found that the levels of VEGF were positively correlated to insulin requirements in patients with T1D. However, we found no correlation to current plasma glucose levels. Previous studies have shown that the plasma levels of VEGF are not acutely affected by blood glucose levels or exogenous insulin infusion [24]. However, the expression of VEGF in vascular smooth muscle cells increases in response to increased levels of glucose [10, 11]. In vascular smooth muscle cells isolated from both humans and insulin sensitive rats it has been demonstrated that insulin also increases the secretion of VEGF [25]. However, in vascular smooth muscle cells from insulin resistant rats the VEGF response to insulin was blunted [25]. We also found a positive correlation between the levels of VEGF and BMI for patients with T1D. Among the patients with T1D in our study only *n* = 11 had a BMI ≥ 30 kg/m^2^ (i.e., fulfilled the criteria for obesity) and *n* = 40 had a BMI of 25–30 kg/m^2^ (i.e., were overweight) whereas the remaining *n* = 64 had a normal BMI. The insulin needs (U × 24 h^−1^  × kg^−1^) were similar in all BMI groups (data not shown) which suggest a normal insulin sensitivity in all patients. A link between VEGF and visceral fat accumulation and BMI has previously been described among overweight and obese, but otherwise healthy, individuals [26]. Based on our data and previously published data it seems as if the increased levels of VEGF in T1D can at least in part be explained by increased secretion rates from vascular smooth muscle cells as a response to exogenous insulin stimulation.

We did not observe any difference in plasma VEGF levels when comparing patients with T1D and microalbuminuria to those without microalbuminuria. In a previous report it has been shown that the plasma levels of VEGF are increased in men, but not women, with T1D and nephropathy [13]. In our study we could not observe any difference in the levels of VEGF between men and women with T1D. Since this is a cross-sectional study, we cannot exclude the possibility that the levels of VEGF were increased prior to onset of microalbuminuria. In a prospective study in pediatric T1D patients it was observed that higher serum levels of VEGF were associated with an increased risk of developing microalbuminuria [15]. Furthermore, in a preclinical study it was demonstrated that overexpression of VEGF in the glomeruli leads to hypertrophy, progressive proteinuria, and decreasing GFR [27]. However, the circulating plasma levels of VEGF were unaltered suggesting that it is the local concentrations of VEGF that are of importance for disease development.

We found that the levels of VEGF were similar in patients with T1D and retinopathy when compared to patients without retinopathy. This is in line with a previous report of plasma levels of VEGF in patients with retinopathy [28] whereas a previous study in a pediatric population showed that the serum levels of VEGF are increased in patients with T1D and retinopathy [17]. Although, it should be noted that in the study by Myśliwiec et al. the patients with retinopathy had a longer disease duration and increased HbA1c levels [17]. In addition, we found that the levels of VEGF were similar in patients with T1D and both microalbuminuria and retinopathy when compared to patients without any of these complications.

In summary, we report on increased circulating plasma levels of VEGF in patients with T1D. However, the plasma levels of VEGF do not correlate to either present or historical metabolic control. The circulating plasma levels of VEGF were found to be similar in patients with and without diabetic microvascular complications.

## Figures and Tables

**Table 1:** Descriptive data for all study participants. The healthy controls and patients with type 1 diabetes (T1D) were matched regarding age, gender distribution, and BMI. The fasting levels of plasma glucose and HbA1c were, as expected, increased in the T1D patients. All values are given as mean ± SEM. An unpaired two-tailed *t*-test based on ranks (Mann–Whitney) was used to compare differences between the two groups for data that were not normally distributed (a) and a two-tailed unpaired student's *t*-test was used for normally distributed data (b).

Parameter	Healthy controls	Type 1 diabetes	*p* value
Total number, *n*	45	115	
Male, *n* (%)	20 (44%)	50 (43%)	
Age (years)	37.4 ± 2.7	40.2 ± 1.5	Ns (a)
Age of onset (years)	N/A	14.6 ± 0.8	N/A
Disease duration (years)	N/A	25.6 ± 1.3	N/A
BMI (kg/m^2^)	25.5 ± 0.9	25 ± 0.3	Ns (a)
Fasting p-glucose (mmol/l)	5.5 ± 0.1	10.8 ± 0.5	<0.0001 (a)
HbA1c (mmol/mol, %)	35.9 ± 0.8 (5.4 ± 0.07%)	62.3 ± 1.1 (7.9 ± 0.1%)	<0.0001 (a)
P-cholesterol (mmol/l)	5.3 ± 0.3	4.7 ± 0.1	Ns (a)
P-HDL cholesterol (mmol/l)	1.4 ± 0.06	1.5 ± 0.04	Ns (b)
P-LDL cholesterol (mmol/l)	3.3 ± 0.2	2.6 ± 0.08	<0.001 (b)
P-triglycerides (mmol/l)	1.3 ± 0.1	1.0 ± 0.05	Ns (a)
P-creatinine (*µ*mol/l)	75.1 ± 2.1	78 ± 2.4	Ns (a)
Estimated GFR (ml/min)	91.2 ± 3.4	92.8 ± 2.2	Ns (b)

## References

[1] Livingstone S. J., Levin D., Looker H. C. (2015). Estimated life expectancy in a scottish cohort with type 1 diabetes, 2008–2010. *JAMA*.

[2] The Diabetes Control and Complications Trial Research Group (1993). The effect of intensive treatment of diabetes on the development and progression of long-term complications in insulin-dependent diabetes mellitus. *The New England Journal of Medicine*.

[3] Gubitosi-Klug R. A., Lachin J. M., Backlund J.-Y. C., Lorenzi G. M., Brillon D. J., Orchard T. J. (2016). Intensive diabetes treatment and cardiovascular outcomes in type1 diabetes: the DCCT/EDIC study 30-year follow-up. *Diabetes Care*.

[4] Agardh C.-D., Agardh E., Torffvit O. (1997). The association between retinopathy, nephropathy, cardiovascular disease and long-term metabolic control in type I diabetes mellitus: a 5 year follow- up study of 442 adult patients in routine care. *Diabetes Research and Clinical Practice*.

[5] Deckert T., Poulsen J. E., Larsen M. (1978). Prognosis of diabetics with diabetes onset before the age of thirtyone—II. Factors influencing the prognosis. *Diabetologia*.

[6] Molitch M. E., DeFronzo R. A., Franz M. J. (2003). Diabetic nephropathy. *Diabetes Care*.

[7] Senger D. R., Galli S. J., Dvorak A. M., Perruzzi C. A., Susan Harvey V., Dvorak H. F. (1983). Tumor cells secrete a vascular permeability factor that promotes accumulation of ascites fluid. *Science*.

[8] Carmeliet P., Collen D. (2000). Molecular basis of angiogenesis. Role of VEGF and VE-cadherin. *Annals of the New York Academy of Sciences*.

[9] Tilton R. G., Kawamura T., Chang K. C. (1997). Vascular dysfunction induced by elevated glucose levels in rats is mediated by vascular endothelial growth factor. *Journal of Clinical Investigation*.

[10] Natarajan R., Bai W., Lanting L., Gonzales N., Nadler J. (1997). Effects of high glucose on vascular endothelial growth factor expression in vascular smooth muscle cells. *American Journal of Physiology—Heart and Circulatory Physiology*.

[11] Williams B., Gallacher B., Patel H., Orme C. (1997). Glucose-induced protein kinase C activation regulates vascular permeability factor mRNA expression and peptide production by human vascular smooth muscle cells in vitro. *Diabetes*.

[12] Sone H., Kawakami Y., Okuda Y. (1996). Vascular endothelial growth factor is induced by long-term high glucose concentration and up-regulated by acute glucose deprivation in cultured bovine retinal pigmented epithelial cells. *Biochemical and Biophysical Research Communications*.

[13] Hovind P., Tarnow L., Oestergaard P. B., Parving H.-H. (2000). Elevated vascular endothelial growth factor in type 1 diabetic patients with diabetic nephropathy. *Kidney International, Supplement*.

[14] Chiarelli F., Spagnoli A., Basciani F. (2000). Vascular endothelial growth factor (VEGF) in children, adolescents and young adults with type 1 diabetes mellitus: relation to glycaemic control and microvascular complications. *Diabetic Medicine*.

[15] Santilli F., Spagnoli A., Mohn A. (2001). Increased vascular endothelial growth factor serum concentrations may help to identify patients with onset of type 1 diabetes during childhood at risk for developing persistent microalbuminuria. *Journal of Clinical Endocrinology and Metabolism*.

[16] Seckin D., Ilhan N., Ilhan N., Ertugrul S. (2006). Glycaemic control, markers of endothelial cell activation and oxidative stress in children with type 1 diabetes mellitus. *Diabetes Research and Clinical Practice*.

[17] Myśliwiec M., Balcerska A., Zorena K., Myśliwska J., Lipowski P., Raczyńska K. (2008). The role of vascular endothelial growth factor, tumor necrosis factor alpha and interleukin-6 in pathogenesis of diabetic retinopathy. *Diabetes Research and Clinical Practice*.

[18] Zorena K., Myśliwska J., Myśliwiec M. (2010). Association between vascular endothelial growth factor and hypertension in children and adolescents type I diabetes mellitus. *Journal of Human Hypertension*.

[19] Levey A. S., Bosch J. P., Lewis J. B., Greene T., Rogers N., Roth D. (1999). A more accurate method to estimate glomerular filtration rate from serum creatinine: a new prediction equation. *Annals of Internal Medicine*.

[20] Jelkmann W. (2001). Pitfalls in the measurement of circulating vascular endothelial growth factor. *Clinical Chemistry*.

[21] Schlingemann R. O., Van Noorden C. J. F., Diekman M. J. M. (2013). VEGF levels in plasma in relation to platelet activation, glycemic control, and microvascular complications in type 1 diabetes. *Diabetes Care*.

[22] Walz J. M., Boehringer D., Deissler H. L. (2016). Pre-Analytical parameters affecting vascular endothelial growth factor measurement in Plasma: identifying confounders. *PLoS ONE*.

[23] Bott U., Jorgens V., Grusser M., Bender R., Muhlhauser I., Berger M. (1994). Predictors of glycaemic control in Type 1 diabetic patients after participation in an intensified treatment and teaching programme. *Diabetic Medicine*.

[24] Dullaart R. P. F., Oomen P. H. N., Sluiter W. J. (2007). Circulating vascular endothelial growth factor is unaffected by acute hyperglycemia and hyperinsulinemia in type 1 diabetes mellitus. *European Journal of Internal Medicine*.

[25] Doronzo G., Russo I., Mattiello L., Anfossi G., Bosia A., Trovati M. (2004). Insulin activates vascular endothelial growth factor in vascular smooth muscle cells: influence of nitric oxide and of insulin resistance. *European Journal of Clinical Investigation*.

[26] Miyazawa-Hoshimoto S., Takahashi K., Bujo H., Hashimoto N., Saito Y. (2003). Elevated serum vascular endothelial growth factor is associated with visceral fat accumulation in human obese subjects. *Diabetologia*.

[27] Liu E., Morimoto M., Kitajima S. (2007). Increased expression of vascular endothelial growth factor in kidney leads to progressive impairment of glomerular functions. *Journal of the American Society of Nephrology*.

[28] Chaturvedi N., Fuller J. H., Pokras F., Rottiers R., Papazoglou N., Aiello L. P. (2001). Circulating plasma vascular endothelial growth factor and microvascular complications of type I diabetes mellitus: the influence of ACE inhibition. *Diabetic Medicine*.

